# Health-Related Quality of Life in Advanced Non-small Cell Lung Cancer: A Methodological Appraisal Based on a Systematic Literature Review

**DOI:** 10.3389/fonc.2019.00715

**Published:** 2019-08-12

**Authors:** Lotte Van Der Weijst, Yolande Lievens, Wim Schrauwen, Veerle Surmont

**Affiliations:** ^1^Department of Radiotherapy-Oncology, Ghent University Hospital, Ghent, Belgium; ^2^Department of Medical Oncology, Ghent University Hospital, Ghent, Belgium; ^3^Department of Thoracic Oncology, Ghent University Hospital, Ghent, Belgium

**Keywords:** quality of life, review–systematic, lung cancer, methodological quality, NSCLC

## Abstract

**Background:** The majority of lung cancer patients are diagnosed with advanced non-small cell lung cancer (NSCLC), the bulk of which receive palliative systemic treatment with the goal to provide effective symptom palliation and safeguard health-related quality of life (HRQoL). Advanced NSCLC trials with HRQoL endpoints face methodological constraints limiting interpretability.

**Objectives:** We provide a comprehensive overview of recent clinical trials evaluating the impact of systemic therapies on HRQoL in advanced NSCLC, focusing on the methodological quality, with the ultimate goal to improve interpretation, comparison and reporting of HRQoL data.

**Methods:** A systematic literature review was performed. Prospective studies published over the last decade evaluating the impact of systemic treatments on HRQoL in advanced NSCLC were included. Methodological quality of HRQoL reporting was assessed with the CONSORT-PRO extension.

**Results:** Hundred-twelve manuscripts describing 85 trials met all criteria. No formal conclusion can be drawn regarding the impact on HRQoL of different treatments. We report an important variety in methodological quality in terms of definitions of HRQoL, missing data points, lack of standardization of analyzing and presenting HRQoL and no standard follow-up time. The quality of HRQoL data reporting varies substantially between studies but improves over time.

**Conclusion:** This review shows that in the heterogeneous landscape of trials addressing HRQoL in advanced stage NSCLC. Methodology reporting remains generally poor. Adequate reporting of HRQoL outcome data is equally important to support clinical decision-making as to correctly inform health policy regarding direct approval and reimbursement of the new drugs and combinations that will come online.

## Introduction

Lung cancer contributes to 1.6 million deaths a year, making it the deadliest cancer worldwide (1). Approximately 80% of primary lung cancers are non-small cell lung cancer (NSCLC) (2). Locally advanced (stage IIIB) or metastatic (stage IV) NSCLC is diagnosed in most patients (65%), leading, despite the development of novel systemic therapies, to poor 5-years survival rates of 5 and 1%, respectively. Palliative therapy aims to prolong survival and to offer acceptable quality of life (QoL). The latter is especially important because besides poor survival, patients with advanced NSCLC also frequently suffer from high symptom burden and toxic therapeutic side effects (3).

Health-related quality of life (HRQoL) has become an integral endpoint in clinical trials for advanced cancer (4, 5). HRQoL is a multi-dimensional concept that addresses the functional effect of a health status and/or a patient's treatment. It embodies physical, role, emotional, social, cognitive, sexual and spiritual functioning on individual levels (6–8). HRQoL data enables treatment comparisons, supports daily clinical treatment decision-making, improves communication between patients and treating clinicians and facilitates clinical and economic evaluations to define the most efficient allocation of healthcare resources (3). Patient-reported HRQoL data particularly aids clinicians to better understand toxicity and symptoms experienced by patients, as subjective symptoms, such as fatigue and pain are frequently under-reported (9). Finally, baseline HRQoL is an independent predictive value for therapy response and survival (5, 10–13), performing better than certain classic endpoints, such as performance status (5). In order to guide treatment decision making and health policy, qualitative reporting and analysis of HRQoL outcomes are essential. Methodological flaws might lead to inaccurate interpretation of outcomes and hinder implementation in clinical practice. Correspondingly, poor data quality and methodological concerns have limited the influence of HRQoL data on the Food and Drug Administrations' regulatory decision making process (3). Methodological constraints in HRQoL data reporting and analysis have been previously highlighted (5).

A number of systematic literature reviews have already been conducted on clinical trials using HRQoL endpoints in patients with advanced NSCLC, all studying a specific question (Table 1). This systematic literature review aims to add to the available evidence by providing a comprehensive overview of all prospective studies published over the last decade, evaluating the impact of various systemic treatments on HRQoL in advanced NSCLC. In addition, the methodological quality of this set of papers is analyzed with the ultimate goal to discuss challenges in and recommendations for the interpretation and comparison of HRQoL evidence obtained from randomized-controlled trials (RCTs).

**Table 1:** Overview of systematic literature reviews of HRQoL in advanced NSCLC trials published since 2007 in chronological order.

**References**	**Title**	**Purpose**	**Time frame**	**Conclusions**	**Limitations**
Tanvetyanon et al. (14)	A systematic review of quality of life associated with standard chemotherapy regimens for advanced non-small cell lung cancer.	To evaluate the effect of standard chemotherapy regimens.	January 1966–May 2006	No large varieties between chemotherapies have been found. HRQoL outcome comparisons are hardly feasible due to heterogeneity and low compliance to HRQoL evaluations.	Focuses solely on chemotherapy. Only 13 articles are included.
Pat et al. (15)	Systematic review of symptom control and quality of life in studies on chemotherapy for advanced non-small cell lung cancer: how CONSORTed are the data?	To evaluate compliance to the CONSORT checklist in RCT comparing chemotherapy.	1980–August 2005	Compliance to CONSORT is reasonable. Large differences between journals and no improvements were found.	Focuses solely on chemotherapy.
Claassens et al. (16)	Health-related quality of life in non-small-cell lung cancer: an update of a systematic review on methodologic issues in randomized controlled trials.	To evaluate HRQoL measurements	2002–2010	Incorporation of HRQoL endpoints has increased. Quality of HRQoL methodology reporting has improved, however specific domains remain inadequately reported.	Focuses on the qualitative aspects of HRQoL reporting.
Matsuda et al. (17)	Quality of life in advanced non-small cell lung cancer patients receiving palliative chemotherapy: a meta-analysis of randomized controlled trials.	To provide an overview of HRQoL in chemotherapy trials.	Until April 2010	Carboplatin-based chemotherapy is associated with better global QoL than cisplatin-based chemotherapy.	Focuses on the comparison of carboplatin- to cisplatin-based chemotherapy. Only 6 trials are included.
Ganguli et al. (18)	The impact of second-line agents on patients' health-related quality of life in the treatment for non-small cell lung cancer: a systematic review.	To assess HRQoL in second-line treatment trials.	2000–2010	Improvement in overall QoL were inconsistent. Large varieties in methodology hinders comparisons.	Focuses solely on second-line chemotherapy.
Saad et al. (19)	Assessment of quality of life in advanced non-small-cell lung cancer: an overview of recent randomized trials.	To provide an overview of trials with HRQoL endpoints.	1997–2009	The majority of trials incorporate HRQoL endpoint. Almost half of included trials reported a significant difference.	Articles published only in 13 leading journals.
Damm et al. (3)	Health-related quality of life questionnaires in lung cancer trials: a systematic literature review.	To evaluate the HRQoL measurements used in trials.	2001–2011	Wide range of HRQoL questionnaires was used. The EORTC QLQ-C30 and its lung cancer specific module was the most frequently used.	Focuses mainly on HRQoL questionnaires.
Mannion et al. (20)	Effect of chemotherapy on quality of life in patients with non-small cell lung cancer.	To evaluate HRQoL in palliative chemotherapy trials.	1987–2011	QoL is an important outcome in advanced NSCLC.	Focuses solely on chemotherapy.
Fiteni et al. (21)	Methodology of health-related quality of life analysis in phase III advanced non-small-cell lung cancer clinical trials: a critical review.	To evaluate the methodology of HRQoL analysis and reporting in phase III first-line chemotherapy trials.	2008–2014	Shortcomings and heterogeneity in measurement, analysis and reporting of HRQoL. Comparisons of HRQoL between trials remains therefore difficult.	Focuses on the methodology of HRQoL analysis in phase III trials of first-line chemotherapy.
Bouazza et al. (22)	Patient-reported outcome measures (PROMs) in the management of lung cancer: a systematic review.	To describe and compare available PROMs.	January 2010–February 2016	PROMs are important in clinical practice if introduced adequately.	Focuses on PROMs rather than HRQoL.

## Materials and Methods

### Literature Search

**Figure 1:**
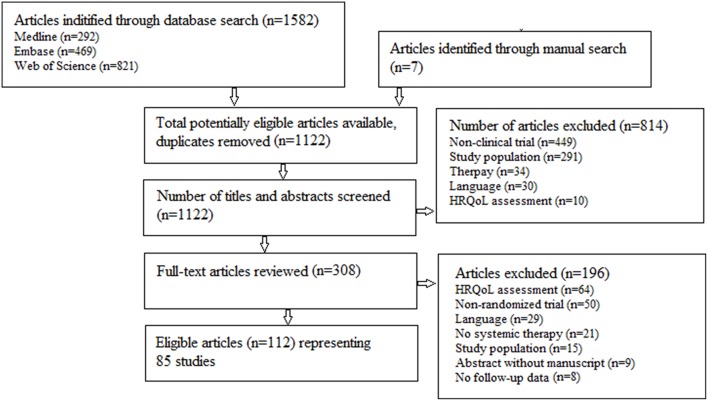
Flow chart describing the selection of eligible manuscripts.

A literature search, following the PRISMA principles (23), was performed in Medline, Web of Science and Embase with both systematic and free text terms concerning HRQoL, advanced and NSCLC (Figure 1). The last search was performed May 29, 2018.

### Study Selection

The search focuses on articles published between January 2007 and December 2017, including those electronically available within this period. Non-randomized trials, pilot and retrospective studies, abstracts without manuscript and non-English articles were excluded. Articles were eligible if the study population included patients with stage IIIB and/or IV NSCLC receiving palliative systemic therapy and measuring HRQoL for more than one time points. Secondary articles, covering the same study population as the primary article, were included and combined into the data synthesis, provided they added information concerning HRQoL.

### Data Extraction

Study selection was two-staged. Firstly, title and abstract screening against selection criteria was undertaken by a single reviewer (LVDW). The other authors were consulted for disagreement resolution. Secondly, full-text articles were extracted based on the eligibility criteria to select the final sample of studies.

### Methodological Quality

The quality of HRQoL data reporting of the included studies was assessed with the CON-solidated Standards of Reporting Trials (CONSORT)—Patient-Reported Outcomes (PRO) checklist (24). The five PRO-specific minimum recommendations for reporting randomized controlled trials were used to assess quality of reporting.

Cut-off scores for reporting quality are: “good” ≥80%; “moderate” 50–79%; and “poor” ≤ 49% (24). In case items were not applicable, such as allocation concealment mechanism, they were excluded in the total outcome. In case secondary manuscripts were published on HRQoL outcomes, both the primary and secondary manuscript were included in the quality assessment.

## Results

Eleven hundred and twenty-two abstracts were identified for initial review. A total of 308 articles underwent in-depth analysis. Of these, 112 articles representing 85 trials were included in this review. The review process is summarized in Figure 1.

### Study Characteristics

In brief, 34,897 patients were enrolled in 85 RCTs, ranging from 37 to 1,433 patients per study. Most of the identified literature presented phase III (*n* = 59; 69%) RCTs. Fifty-nine studies (69%) analyzed first-line therapy. The most frequently used primary (co-) endpoint was survival (*n* = 34; 40%), followed by progression free survival (PFS) (*n* = 32; 38%), response rate (*n* = 9; 11%) and QoL (*n* = 6; 7%).

In total, 41 studies compared different chemotherapies, 4 studies compared different targeted therapies. Twenty studies compared chemotherapy with targeted therapy; 1 with immunotherapy. Respectively, 14 and 1 studies compared targeted and chemotherapy to placebo. Four studies compared different sequential therapies consisting of both targeted therapy and chemotherapy. A brief synopsis of the main study characteristics is presented in the Supplementary Material.

**Figure 2:**
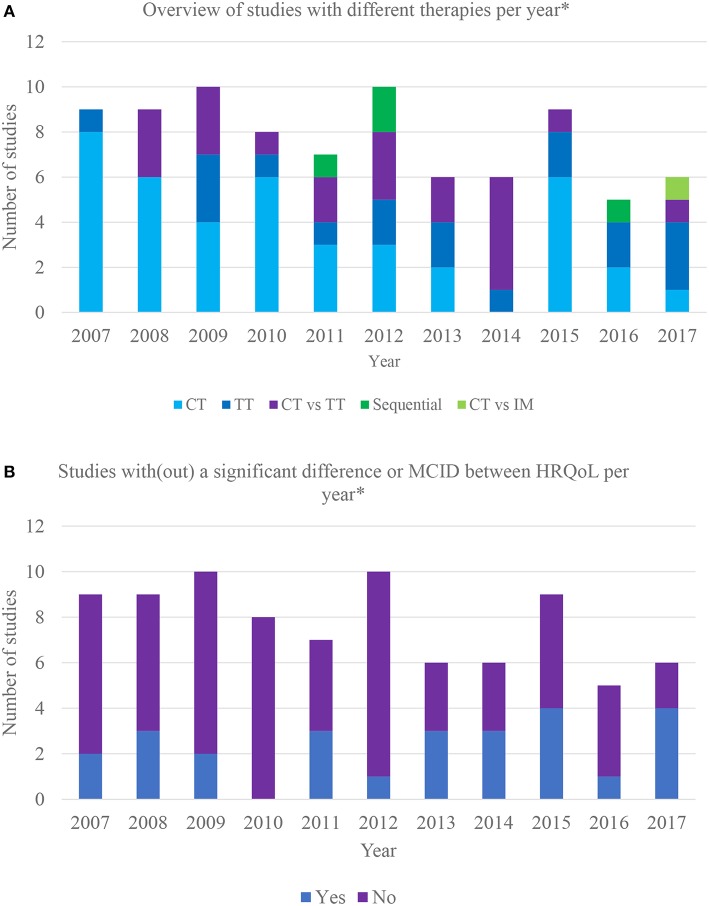
**(A)** Overview of different therapies incorporated in clinical trials over the last 10 years. *Based on the year of HRQoL publication; CT, chemotherapy vs. chemotherapy or placebo; TT, targeted therapy vs. targeted therapy or placebo; CT vs. TT, chemotherapy vs. targeted therapy; sequential: sequential therapy consisting of both targeted and chemotherapy; CT vs. IM, chemotherapy vs. immunotherapy. **(B)** Overview of clinical trials with/without significant differences in HRQoL between therapy arms over the last 10 years. *Based on the year of HRQoL publication; MCID, meaningful clinically important difference.

Figure 2A provides an overview of studies incorporating different therapies per year, illustrating that the interest for the HRQoL impact of systemic treatments progressively shifted from chemotherapy to chemotherapy vs. targeted therapy and targeted therapy vs. placebo.

### Impact of Systemic Treatments on HRQoL

Twenty-six (31%) studies found a statistically or minimal clinically important difference (MCID) in HRQoL between therapy arms. Furthermore, some reported a difference in certain domains or symptoms. MCID refers to smallest change in an outcome that is important to the patient (25). Respectively, 22% (*n* = 9) (26–36) and 50% (*n* = 2) (37–39) of the trials assessing the impact on HRQoL of various chemotherapeutic combinations and different targeted therapies reported a difference. The only trial (40, 41) comparing chemotherapy to placebo reported no difference, only 2 out of 14 studies showed that targeted therapy causes a positive impact on HRQoL compared to placebo (42–44). Eleven studies (55%) comparing chemo- with targeted therapy reported a difference (45–60), with targeted therapy favored in nine. One study reported that gefitinib is favored in patients with EGFR mutations and carboplatin/paclitaxel in those without mutations (61, 62). The only study comparing chemotherapy to immunotherapy favored the latter (63, 64). One out of 4 sequential therapy RCTs reported a difference between arms (65, 66). The results are summarized in Figure 2B.

### Quality of HRQoL Data

Eighteen different HRQoL measurement tools were used, including generic, cancer-, lung cancer-, and symptom specific tools. The cancer-specific European Organization for Research and Treatment of Cancer Quality of Life Questionnaire (EORTC QLQ-C30) alone or together with its lung cancer-specific EORTC QLQ-LC13 supplementary module was the dominantly used HRQoL instrument (*n* = 38; 45%). The Functional Assessment of Cancer Therapy-Lung (FACT-L) questionnaire was employed 27 (32%) times and the lung cancer symptom scale (LCSS) tool was used in 11 (13%) trials.

HRQoL data analysis is based on a large variety of statistical techniques, ranging from Mann–Whitney *U* test (*n* = 11; 13%), logistic regression model (*n* = 8; 9%) to *t*-test (*n* = 6; 7%). Twenty-three studies lacked clarification on statistical methods applied.

**Figure 3:**
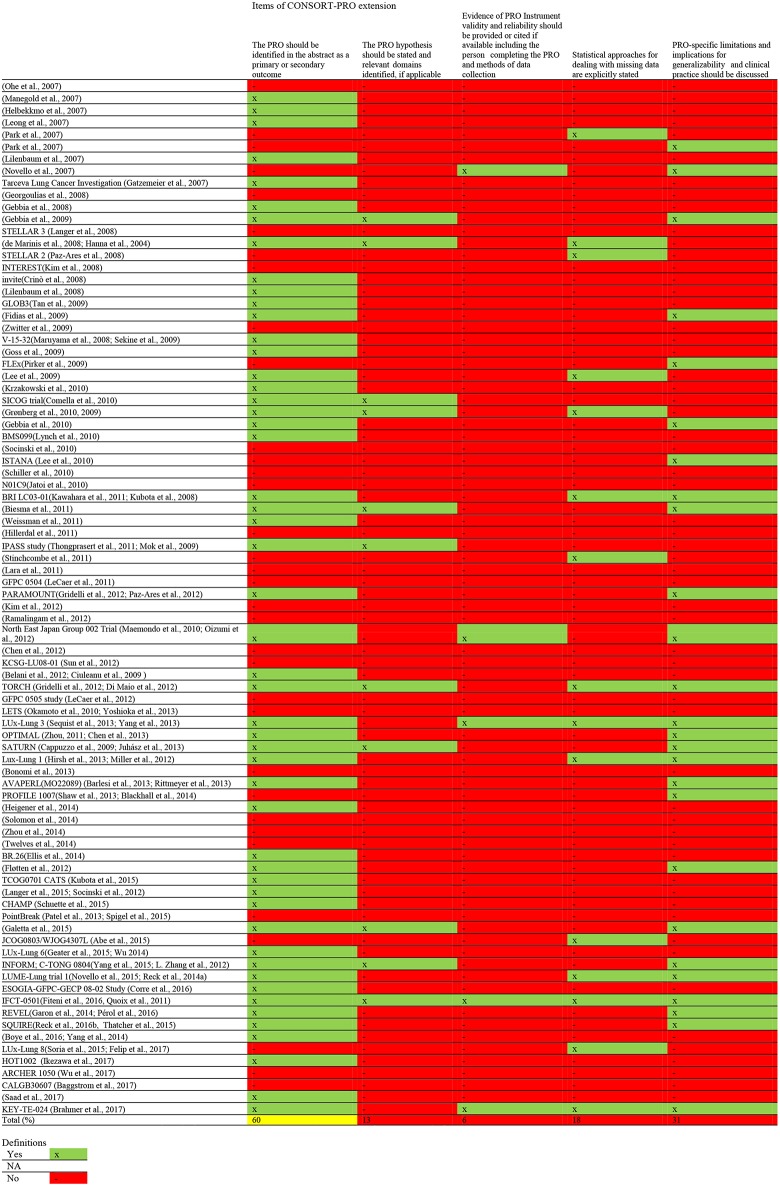
Overview of the quality aspects of PROs of each individual studies.

The quality of HRQoL reporting is summarized in Table 2. Figure 3 provides a comprehensive overview of the quality aspects concerning PROs of each individual study. All PRO items were scored poorly, except for identification PRO as a primary or secondary outcome in the abstract which was scored moderate. Only one study fulfilled all criteria (67, 68).

**Table 2:**
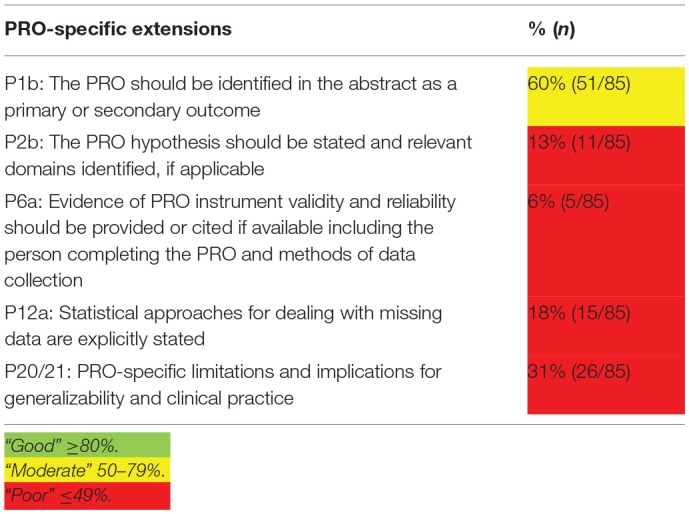
Summary of quality of HRQoL reporting.

## Discussion

A number of systematic literature reviews on HRQoL in advanced NSCLC trials have already been conducted (Table 1), all focusing on specific treatments or aspects. This review addresses the entire spectrum of nowadays' systemic treatment strategies, newer drugs as well as chemotherapy, in trials published over the last 10 years. In addition, in contrast to the other reviews, it provides both a synopsis of study characteristics and methodological quality of HRQoL reporting.

Maintenance and improvement of HRQoL in advanced NSCLC as a result of treatment is important due to the limited impact which therapies have on prolonging life. A qualitative study on chemotherapy preferences reported that advanced NSCLC patients favor chemotherapy over best supportive care in case HRQoL improves with chemotherapy, even without improving survival (69). Accordingly, the Food and Drug Administration started approving new drugs, such as erlotinib, based on the combination of longer PFS with positive impact on HRQoL or other PROs, without significantly improving overall survival. To-date however, the patient perspective is not yet fully embedded in drug approval, and when PROs are used, they are typically limited to improvement in symptoms rather than HRQoL (70). It has however been shown that PROs are more strongly associated with measures of daily health status than clinician assessment (71). High quality patient-reported HRQoL data may therefore provide meaningful information for risk-benefit evaluation and should be used to support drug approval and reimbursement policy. In this review, 85 studies on advanced NSCLC with HRQoL end-points were identified, enrolling almost 35,000 patients. Despite these large numbers, the vast heterogeneity of agents used does not allow one to draw overall conclusions, nor on the actual HRQoL gains, nor on the most effective drugs to improve HRQoL. Based on the study results, targeted therapies seem to be favored over chemotherapy in terms of HRQoL, but interpretation on a case by case basis remains necessary. One review concluded that carboplatin-based chemotherapy is associated with better global QoL compared to cisplatin-based regimes; no other conclusions were drawn in the reviews (17).

Although, only one immunotherapy RCT has been included in this review, immunotherapy is an emerging and continuously evolving field within thoracic oncology. The first data on HRQoL from immunotherapy trials is now appearing. These initial results show that HRQoL is maintained and improved more significantly with immunotherapy compared to chemotherapy (72–75). More data on HRQoL in immunotherapy is expected in the near future.

In addition to the actual outcome data supporting clinical decision-making, consistency in collecting, analyzing and reporting HRQoL data is important to guide health policy. Especially in an era where new, expensive and potentially toxic drug combinations are frequent. HRQoL data may have an impact on reimbursement policies. Previous studies have highlighted the multidimensional nature of HRQoL, diversity in research questions, lack of a priori hypothesis, repeated measures and high likelihood of missing data which hampers drawing meaningful conclusions (76). Standardization—in HRQoL end-points included in trials, in analysis and reporting of HRQoL data—is, therefore, needed. Paucity of standardization limits the interpretability and comparability of HRQoL data between therapies and has implications on decision-making (3). The Setting International Standards in Analyzing Patient-Reported Outcomes and Quality of Life Endpoints Data (SISAQOL) consortium has been established to develop recommendations on analysis and interpretation of PROs in oncology trials (76). When designing an oncology trial with HRQoL endpoints and interpreting HRQoL data, in the future it will be important to consult the recommendations of the SISAQOL. Furthermore, HRQoL results should be presented according to the CONSORT-PRO checklist, aiming to facilitate optimal reporting of PRO data in RCTs (24). Several HRQoL data capturing tools are used, including generic, condition- and symptom-specific questionnaires. Outcome data derived from these questionnaires differ substantially in terms of HRQoL parameters and lung cancer-associated symptoms (3). Montazeri et al. recommends using the EORTC QLQ-C30 questionnaire and its lung cancer-specific module (QLQ-LC13) in lung cancer trials (77, 78). However, since the introduction of the QLQ-LC13 in 1994, major developments have occurred in the diagnosis and treatment of lung cancer. The EORTC has recognized this challenge and has therefore updated this questionnaire (QLQ-LC29), encompassing the impact of new and innovative treatment strategies on HRQoL (79).

The EORTC QLQ C30 is used in almost half of the analyzed studies. Yet inter-comparison among studies remains difficult as some selectively report certain domains or symptoms of HRQoL, for example an improvement in cough, pain, dyspnea and physical functioning using erlotinib, without mentioning the remaining HRQoL parameters (80). Other studies solely report significant differences between treatment arms, but lack reporting on whether HRQoL is maintained, improved or whether it deteriorates (81–83). Other studies report percentile symptom improvements or the mean HRQoL deterioration time (55), moreover, the majority of RCTs stop HRQoL measurement some months after end of therapy or solely rely on HRQoL evaluations during and at the end of treatment, not capturing long-term effects. Although toxicities associated with targeted and chemotherapy regimens, influencing HRQoL, may be either reversible or continue to prevail during most of the patient's life, recommendations on duration of the follow-up period are still inexistent (84).

From a statistical point of view, an even larger variation exists. If longitudinal HRQoL data is available, standardized approaches to analyze and define MCID scores are necessary. MCID should be applied, as statistically significant improvement or worsening of HRQoL scores may be too small to be clinically relevant to the patient (85). As mentioned, the lack of standardization hinders drawing conclusions on the clinical meaningfulness of HRQoL data, hence, assisting treatment decisions (3). As a result of these issues, the SISAQOL consortium was established in 2016 (76). Missing multiple data points also remains problematic and seems inevitable in longitudinal HRQoL datasets in the advanced NSCLC population (5). Patients with poor health at baseline typically have poor baseline HRQoL, may have worse disease progression and have a HRQoL that deteriorates faster. These patients generally drop out earlier than patients with better baseline HRQoL. This potentially leads to bias, affects trial results and conclusions, and eventually clinical practice. (68, 86). Accordingly, missing data in repeated measurements over time combined with the multidimensionality of HRQoL data requires statistical analysis techniques capable of dealing with these issues (69).

Qualitative and complete reporting of HRQoL results in scientific articles is crucial to allow applying HRQoL evidence from clinical trials into daily clinical decision-making and treatment policies. Our data demonstrates persisting inadequate presentation of specific domains and overall poor reporting quality, not dissimilar from the observations made in previous reviews (16). The limited methodological quality and lack of certain crucial aspects hinders comparing and interpreting HRQoL data, necessary to support optimal decision-making, problematic.

Apart from the quality issues described, the very nature of clinical trials *per se* may hamper translating evidence into practice. Strict inclusion criteria concerning age and performance status limit the overall generalization of HRQoL data. Of the 85 analyzed studies, only 12 focused particularly on elderly patients, whereas they represent the majority of the lung cancer patient population (35, 46, 47, 49, 67, 68, 87–93). Hence, collecting HRQoL real-life data has added value to determine the best standard of care and to define the most efficient healthcare resource allocation involving patients with advanced NSCLC (20).

Finally, although this review aimed to provide a comprehensive review of the recent peer-reviewed literature, it may have failed to capture potentially important evidence, as only full-text publications were included and studies not published in English excluded. The exclusion of gray literature has nonetheless been applied to guarantee the validity of the included articles in terms of methodological quality (94). Another factor to be considered is that the publication period was restricted to 10 years, in order to capture evidence on the drugs most relevant to date.

## Conclusion

Our systematic review provides a comprehensive overview of recent RCTs evaluating the impact of systemic therapies on HRQoL in advanced NSCLC. It focuses on the methodological quality of these papers.

A vast variety in HRQoL measurements, data collection time points and reporting and analyzing of HRQoL data makes comparisons of outcomes hardly feasible. Quality of reporting HRQoL outcomes remains poor with certain aspects being systematically underreported.

Nevertheless, adequate and complete reporting is critical to inform health policy and clinical decision-making to sustain and improve HRQoL in this critical patient population. This is particularly important since new, expensive and potentially toxic therapies, often in combination, are being introduced.

Future clinical trials exploring novel therapies for advanced NSCLC should focus on reporting HRQoL data in a clinically meaningful and methodologically qualitative way. Additionally, further research should focus on developing standards to optimize and on defining MCID scores.

## Author Contributions

All authors listed have made a substantial, direct and intellectual contribution to the work, and approved it for publication.

### Conflict of Interest Statement

The authors declare that the research was conducted in the absence of any commercial or financial relationships that could be construed as a potential conflict of interest.
