# Supplementary material for: Health-Related Quality of Life in Advanced Non-small Cell Lung Cancer: A Methodological Appraisal Based on a Systematic Literature Review
Source: Front Oncol. 2019 Aug 12;9:715. doi: 10.3389/fonc.2019.00715 (PMC6699450; doi:10.3389/fonc.2019.00715)
Supplement: Supplementary file 1 [file Data_Sheet_1.docx]

Tables of characteristics of included studies.

Table 1. HRQoL in comparing chemotherapy to chemotherapy.

| **Reference (Name study)** | **Treatment outline** | **Nr of patients** | **Primary endpoint** | **HRQoL measurement instrument** | **Analyzing technique** | **Difference in HRQoL outcome** |
| --- | --- | --- | --- | --- | --- | --- |
| (95) | CIS/irinotecan vs. CAR/PAC vs.  CIS/GEM vs. CIS/VIN | 602 | OS | FACT-L; QoL- ACD | Unknown | No, only physical domain was worse in CIS/irinotecan arm |
| (19) | GEM vs. DOC and introduction of the opposite agent in case of PD | 330 | Feasibility | QLQ-C30/LC13; SS14 | Wilcoxon signed-rank test | Yes, favoring GEM followed by DOC |
| (67) | VIN/CAR vs. GEM/CAR | 432 | OS | QLQ-C30/LC13 | Mann–Whitney U-test; two- sided t-test | No |
| (78) | GEM vs. VIN vs. DOC | 134 | Tolerability; ORR; QoL | QLQ-C30/LC13 | Based on guidelines for assessing QoL in EORTC clinical trials | No |
| (20) | 2 vs. 4 additional 3^rd^ generation platinum doublet CT | 452 | RR | QLQ-C30/LC13 | ANCOVA for repeated measures | Yes, favoring 4 additional cycles |
| (96) | CIS+/either 3-weekly vs. weekly DOC | 86 | RR | QLQ-C30 | Paired t-tests | No |
| (86) | DOC every 3 weeks vs. weekly | 111 | Frequency of grade 3/4 toxicity | FACT-L | General linear models | No |
| (97) | 2 cycles of GEM/CIS followed by: 3 additional cycles of GEM +/- CIS | 340 | OS | LCSS | chi-square test | No |
| (22) | DOC/GEM vs. DOC | 312 | OS | LCSS | Pearson’s chi-square test | Yes, favoring DOC/GEM |
| (98) | VIN on day 1,8 and 15+/CIS on day 1 every 4 weeks vs. VIN on day 1 and 8/CIS on day 1 every 3 weeks | 278 | OS; toxicity; therapy compliance | QLQ-C30/LC13 | Unknown | No |
| BRI LC03-01 (99,100) | VIN/GEM followed by DOC vs. PAC/CAR | 401 | OS | FACT-L; FACT- taxane; FACIT-Sp | General linear mixed-effects model | No difference in FACT-L & FACT-Sp data, but QoL is better in PAC/CAR arm in FACT-Taxane |
| STELLAR 3(68) | CAR/PAC vs CAR/PAC Poliglumex (CT-2103, PPX) | 400 | Survival | FACT-LCS | Unknown | No |
| (101,102) | PEM vs. DOC | 571 | OS | LCSS | Kaplan-Meier method | No; both arms improved |
| STELLAR 2 (103) | PAC poliglumex vs DOC | 849 | OS | FACT-LCS | Fisher’s exact test | No |
| DISTAL-2 (69) | DOC vs. DOC/GEM or VIN (depending on 1^st^ line) vs. DOC/capecitabine | 400 | OS | QLQ-C30/LC13 | Wilcoxon rank sum test | No, except of worsening of appetite, vomiting & hemoptysis in DOC/GEM arm |
| GLOB3  (104) | Oral and i.v. VIN/CIS vs. DOC/CIS | 390 | TTF | Global QOL, nausea/vomiting, dyspnoea and pain of QLQ-C30 | Unknown | No |
| (105) | DOC immediately after GEM/CAR vs. DOC at PD after GEM/CAR | 566 | OS | LCSS | Mantel- Haenszel χ 2  test | No |
| (106,107) | CAR/PEM vs. CAR/GEM | 446 | HRQoL | QLQ-C30/LC13 | Linear regression | No |
| (23) | GEM (standard infusion)/CIS vs GEM (prolonged low-infusion)/CIS | 249 | PFS + OS | LCSS + simplified scale for assessment of QoL | Unknown | Yes, favoring  low-dose long infusion group |
| (108) | Vinflunine vs. DOC | 551 | PFS | FACT-L | Unknown | No |
| SICOG trial (109) | GEM/PAC vs. GEM/PEM | 105 | RR, acute toxicity | QLQ-C30/LC13 | Mann–Whitney test | No, except hair loss & perception of neuropathy peripheral was worse in PAC/GEM arm |
| (110) | CIS/DOC vs. CIS/VIN | 86 | QoL, symptom control, side effects | QLQ-C30/LC13 | Wilcoxon rank sum test | No |
| BMS099 (111) | CET/taxane (DOC or PAC) vs. CET/CAR | 676 | PFS | FACT-L | Unknown | No |
| (112) | PEM/CAR +/- Enzastaurin vs. DOC/CAR | 228 | TTP | FACT-L; FACT-Taxane | Unknown | No |
| (79) | CAR/GEM vs. CAR/PAC | 182 | Change in global QoL | QLQ-C30/LC13 | Mann–Whitney test | No |
| (24) | GEM/oxaliplatin vs. CAR/PAC | 383 | Efficacy tolerability | FACT-L | Unknown | Yes, favoring GEM/oxaliplatin; both arms improved |
| (113) | GEM/CAR +/- sequential DOC | 131 | TTP | QLQ-C30/LC13 | Unknown | Global health remained stable; nausea & vomiting increased & pain, dyspnoea, chest pain, hemoptysis & couching decreased in both arms. Peripheral neuropathy & alopecia increased in DOC arm |
| IFCT-0501 (80,81) | CAR/PAC vs VIN vs GEM | 451 | OS | QLQ-C30/LC13 | Kaplan–Meier estimation; log-rank test | No |
| (114) | VIN/GEM vs. VIN/CAR | 444 | OS | QLQ-C30/LC13 | Mann–Whitney U-test. | No |
| PARAMOUNT  (115,116) | PEM/CIS + followed by maintenance PEM/BSC vs PEM/CIS followed by BSC/placebo | 539 | PFS | EQ-5D | Mixed-effects analysis of variance model | No |
| AVAPERL(MO22089)(117,118) | BEV/CIS/PEM followed by maintenance BEV vs. BEV/PEM | 414 | PFS | QLQ-C30/LC13 | Unknown | No |
| (119) | CET/BEV (6 cycles) + PAC/CAR 3 vs. 6 cycles | 121 | PFS | FACT-L | Wilcoxon rank sum test | No |
| (25,26) | Albumin-bound PAC+CAR (nab-PC) vs. solvent-based PAC/CAR | 546 | ORR | FACT-Taxane | Two-sample t test | Yes, favoring Nab-PC |
| PointBreak (21,29) | PEM/CAR/BEV followed by maintenance PEM/BEV vs. PAC/CAR/BEV followed by maintenance BEV | 939 | OS | FACT-L; FACT-Ntx | Linear mixed-effects models | No, except for neurotoxicity favoring PEM arm & fatigue, favoring PAC arm |
| LETS (120,121) | CAR/S-1 vs CAR/PAC | 564 | OS | FACT-LCS; neurotoxicity subscale of FACT/GOG-Ntx | Linear mixed-effects models | No difference in LCS scale. Scores on neurotoxicity scale increased in CAR/PAC arm after 2 cycles. Alopecia was worse in CAR/PAC |
| TCOG0701 CATS (27) | S-1/CIS vs. DOC/CIS | 608 | OS | QLQ-C30/LC13 | Unknown | Yes, favoring S-1/CIS |
| CHAMP  (122) | Panitumumab/PEM/CIS vs. PEM/CIS | 98 | PFS | QLQ-C30/LC13 | Repeated measures ANOVA | No |
| GOIM ERACLE  (123) | CIS/PEM, followed by PEM vs. CAR/PAC/BEV followed by BEV | 118 | QoL | EQ-5D | Students t-test; Wilcoxone Manne Whitney; linear model | No |
| JCOG0803/WJOG4307L (28) | DOC +/- CIS | 276 | OS | FACT-LCS | repeated measures ANOVA | HRQoL decline in DOC/CIS arm; remained stable in DOC arm |
| ESOGIA-GFPC-GECP 08-02 Study (82) | Allocation on basis of PS and age (CAR-based doublet if PS ≤1 and ≤75; DOC if PS 2 or >75y) vs treatment allocation on basis of CGA (CAR-based doublet for fit patients, DOC for the vulnerable, BSC for frail patients) | 494 | Treatment failure free survival | EQ-5D | linear mixed-effects model | No |
| (124) | GEM/CAR vs GEM/CIS | 71 | Radiological response; toxicity; PFS; OS; QoL | FACT-L | Paired t-test | No |

Table 2. HRQoL in comparing targeted therapy to targeted therapy.

| **Reference** | **Treatment outline** | **Nr** | **Primary endpoint** | **HRQoL measurement instrument** | **Analyzing technique** | **Difference in HRQoL outcome** |
| --- | --- | --- | --- | --- | --- | --- |
| (125) | ERL vs GEF | 96 | RR | QLQ-C30/LC13 | Unknown | No |
| (126) | DAC vs ERL | 188 | PFS | QLQ-C30/LC13 | Unknown | Diarrhea, mucositis & skin toxicity were more common with DAC |
| LUX-Lung 8 (30,31) | AFA vs ERL | 795 | PFS | QLQ-C30/LC13 | Proportional hazards regression; log-rank test | Yes, favoring AFA |
| ARCHER 1050 (32) | DAC vs GEF | 452 | PFS | EORTC QLQ-C30/LC13; EQ-5D | Repeated-measures mixed-effects modelling; Kaplan-Meier method; Hochberg-adjusted log-rank test | Yes, favoring GEF, although small |

Table 3. HRQoL in comparing targeted therapy to chemotherapy.

| **Reference** | **Treatment outline** | **Nr** | **Primary endpoint** | **HRQoL measurement instrument** | **Analyzing technique** | **Difference in HRQoL outcome** |
| --- | --- | --- | --- | --- | --- | --- |
| INTEREST (38) | DOC vs. GEF | 1433 | OS | FACT-L | multivariate logistic regression model | Yes, favoring GEF |
| Invite (39) | GEF vs. VIN | 196 | PFS | FACT-L | logistic regression model | Yes, favoring GEF |
| V-15-32 (46,47) | DOC vs. GEF | 490 | OS | FACT-L | logistic regression model | Yes, favoring GEF |
| (127) | ERL vs. CAR/PAC | 103 | PFS | QLQ-LC13 | Unknown | No |
| North East Japan Group 002 Trial (49,128) | CAR/PAC vs. GEF | 148 | PFS | The Care Notebook | Kaplan–Meier curves; log-rank test | Yes; QoL was maintained longer in patients with GEF than those receiving CAR/PAC |
| ISTANA (129) | DOC vs. GEF | 161 | PFS | FACT-L | χ2 test | No |
| (130) | PEM +/- matuzumab | 148 | ORR | LCSS | Fisher’s exact test | No |
| IPASS study  (54,55) | GEF vs. CAR/PAC | 1217 | PFS | FACT-L | Logistic regression; Kaplan-Meier plots | Yes, favoring GEF in patients with EGFR positive mutation; favoring CAR/PAC in EGFR negative mutation patients |
| OPTIMA/CTONG-0802(50,51) | ERL vs. GEM/CAR | 165 | PFS | FACT-L | Logistic regression analysis | Yes, favoring ERL |
| (84) | GEM vs. ERL vs. GEM/ERL | 146 | 6-months PFS | FACT-L | Mixed effects model; χ2 test | No |
| (77) | VIN vs. ERL | 116 | RR | FACT-L | Unknown | No, except physical wellbeing favoring ERL |
| KCSG-LU08-01 (131) | GEF vs. PEM | 135 | PFS | QLQ C30 | ANCOVA | No, except dyspnea & diarrhea favoring GEF & PEM respectively |
| LUX-Lung 3(52,53) | AFA vs. CIS/PEM | 345 | PFS | QLQ-C30/LC13 | Kaplan-Meier plots; Cox proportional hazards regression model; mixed-effects growth  curve models; piecewise linear model | Yes, favoring AFA |
| PROFILE 1007 (40,41) | CRI vs. CT (PEM or DOC) | 347 | PFS | QLQ-C30/LC13 | Repeated measures mixed-effects analyses | Yes, favoring CRI |
| (42) | ERL vs. CAR/VIN | 284 | PFS | FACT-L | Unknown | Yes, favoring CAR/VIN |
| (43) | CRI vs. PEM/CAR or CIS | 343 | PFS | QLQ-C30/LC13; EQ-5D | Repeated-measures mixed-effects modeling; two-sided paired t-test | Yes, favoring CRI |
| (132) | GEF vs. PEM | 161 | PFS | FACT-L | logistic regression analysis | No |
| (133) | PAC/CAR + BEV vs. axitinib | 118 | PFS | QLQ-C30 | Descriptive  analyses | No |
| LUX-Lung 6 (44,45) | AFA vs. GEM/CIS | 364 | PFS | QLQ-C30/LC13 | Kaplan-Meier plots; Cox proportional-Hazards model; mixed-effects growth curve model | Yes, favoring AFA |
| HOT1002 (134) | S-1 vs ERL | 37 | DCR | FACT-L | Unknown | No |

Table 4. HRQoL in comparing immunotherapy to chemotherapy.

| **Reference** | **Treatment outline** | **Nr** | **Primary endpoint** | **HRQoL measurement instrument** | **Analyzing technique** | **Difference in HRQoL outcome** |
| --- | --- | --- | --- | --- | --- | --- |
| KEYNOTE-024 (56,135) | Pembrolizumab vs platinum-doublet CT | 305 | PFS | EORTC QLQ-C30/LC13; EQ-5D-3L | Kaplan-Meier plots, log-rank tests stratified for randomisation, and Cox proportional hazards models | Yes, favoring Pembrolizumab |

Table 5. HRQoL in comparing targeted therapy to placebo.

| **Reference** | **Treatment outline** | **Nr** | **Primary endpoint** | **HRQoL measurement instrument** | **Analyzing technique** | **Difference in HRQoL outcome** |
| --- | --- | --- | --- | --- | --- | --- |
| Tarceva Lung Cancer Investigation (136) | ERL/CIS/GEM vs. placebo/CIS/GEM | 1172 | OS | Time to symptomatic progression (QoL) | Two-sided log-rank  test | No |
| (137) | GEF vs. placebo | 201 | PFS | FACT-L | Logistic regression model | No; improvements in both arms |
| FLEX  (138) | CET/CIS/VIN vs. placebo /CIS/VIN | 585 | OS | QLQ-C30/LC13; EQ-5D | Unknown | No |
| (139) | Thalidomide/CAR/GEM vs placebo /CAR/GEM | 722 | OS | QLQ-C30/LC14 | Repeated measure analysis | No |
| N01C9(85) | Infliximab/DOC vs. placebo/DOC | 61 | ≥10% in weight gain | FAACT | Wilcoxon rank sum tests | No; but lower levels of functional physical wellbeing in DOC/Infliximab arm |
| (140) | Vadimezan/CAR/PAC vs placebo/CAR/PAC | 1299 | OS | QLQ-C30 | Unknown | No; decrease in physical functioning in both arms at end of therapy |
| SATURN  (141,142) | ERL vs. placebo | 889 | PFS | FACT-L | Kaplan–Meier survival; two-sided log-rank tests | No |
| LUX-Lung 1  (35,36) | AFA vs. placebo | 585 | OS | EQ-5D; QLQ-C30/LC13 | χ2 square test; log-rank test stratified; mixed-effects growth-curve model; piecewise linear model | Yes, favoring AFA |
| INFORM; C-TONG 0804  (143,144) | GEF vs placebo | 296 | PFS | FACT-L | Logistic regression model | No |
| BR.26  (145) | DAC vs. placebo | 480 | OS | QLQ-C30/LC13 | Logrank test adjusted by the Hockberg method | No, but hemoptysis in DAC was better & appetite loss, sore mouth & trouble swallowing were worse |
| REVEL  (146,147) | Ramucirumab/DOC vs placebo/DOC | 1253 | OS | LCSS | Kaplan-Meier method; Cox regression | No |
| LUME-Lung trial 1  (148,149) | Nintedanib/DOC vs placebo/DOC | 655 | PFS | QLQ-C30/LC13; EQ-5D | Log-Rank test; mixed-effects growth curve models; piecewise linear model; | No |
| SQUIRE  (150,151) | CIS/GEM +/- Necitumumab | 1093 | OS | LCSS | Kaplan-Meier method; unstratified log-rank tests | No; HRQoL decline in both arms equally |
| CALGB 30607 (37) | Sunitinib vs placebo | 210 | PFS | EORTC QLQ-C30/LC13; EQ-5D | Unknown | Yes, favoring placebo |

Table 6. HRQoL in comparing chemotherapy to placebo.

| **Reference** | **Treatment outline** | **Nr** | **Primary endpoint** | **HRQoL measurement instrument** | **Analyzing technique** | **Difference in HRQoL outcome** |
| --- | --- | --- | --- | --- | --- | --- |
| (33,34) | PEM vs. placebo | 663 | OS | LCSS | Two-sample t-tests | No, except for increase in loss of appetite & delayed worsening of pain & hemoptysis in PEM arm |

Table 7. Sequential therapy.

| **Reference** | **Treatment outline** | **Nr** | **Primary endpoint** | **HRQoL measurement instrument** | **Analyzing technique** | **Difference in HRQoL outcome** |
| --- | --- | --- | --- | --- | --- | --- |
| GFPC 0504 (76) | DOC/GEM followed by ERL after progression vs. ERL followed by DOC/GEM after progression | 100 | Time to 2^nd^ progression | Spitzer index | Unknown | No; slight decrease of HRQoL after therapy in both arms |
| TORCH  (152,153) | ERL followed by CIS/GEM vs. CIS/GEM followed by ERL | 630 | OS | QLQ-C30/LC13 | Exact linear rank test; Gray method | No |
| GFPC 0505(154) | GEM followed by ERL vs  ERL followed by GEM | 100 | Time to 2^nd^ progression | Spitzer index/LCSS | Fisher’s exact test | No; slight decrease of HRQoL after therapy in both arms |
| (58,59) | PEM/CIS followed by maintenance GEF vs. GEF monotherapy | 236 | PFS | LCSS | Kaplan-Meier estimator | Yes, favoring GEF monotherapy |

Abbreviations: III, stage III; IV, stage IV; AE, adverse events; AFA, afatinib; ALK, anaplastic lymphoma kinase; BEV, bevacizumab; BFI, Brief Fatigue Inventory; BSC, best supportive care; CAR, carboplatin; CET, cetuximab; CGA, Comprehensive Geriatric Assessment; CIK, cytokine-induced killer; CIS, cisplatin; CRI, crizotinib; CT, chemotherapy; DAC, dacomitinib; DOC, docetaxel; EGFR, Estimated Glomerular Filtration Rate; EORTC, European Organization for Research and Treatment of Cancer; ERL, erlotinib; GEM, gemcitabine; FAACT, Functional Assessment of Anorexia/Cachexia; FACIT-S, Functional Assessment of Chronic Illness Therapy-Spirituality Subscale; FACT/GOG-Ntx, FACT/Gynecology Oncology Group-Neurotoxicity; FACT-L, Functional Assessment of Cancer Therapy–Lung; FACT-LCS, Functional Assessment of Cancer Therapy–Lung Cancer Subscale; FACT-Taxane, Functional Assessment of Cancer Therapy-taxane; GEF, gefitinib; HRQoL, health-related quality of life; KPS, Karnofsky Performance Status; LCSS, Lung Cancer Symptom scale; nr, number; ORR, overall response rate; OS, overall survival; PAC, paclitaxel; PD, progressive disease; PD-L1, Programmed death-ligand 1*;* PEM, pemetrexed; PFS, progression-free survival; PS, performance status; QoL, quality of life; RR, response rate; RT, radiotherapy*;* TTF, time to treatment failure; TTP, time to progression; VIN, vinorelbine; wet, with pleural effusion; y, years
