# Supplementary material for: Health-Related Quality of Life in Advanced Non-small Cell Lung Cancer: A Methodological Appraisal Based on a Systematic Literature Review
Source: Front Oncol. 2019 Aug 12;9:715. doi: 10.3389/fonc.2019.00715 (PMC6699450; doi:10.3389/fonc.2019.00715)
Supplement: Supplementary file 2 [file Data_Sheet_2.docx]

Medline (via PubMed interface) search May 29, 2018

#1 “Quality of life”[Mesh] OR “*quality of life”[tiab] OR ”Life quality”[tiab]

#2 “squamous cell carcinoma*”[tiab] OR “squamous cell lung carcinoma*”[tiab] OR “non-small cell lung cancer”[tiab] OR “lung adenocarcinoma*”[tiab] OR “non-small cell carcinoma*”[tiab] OR “adenocarcinoma of the lung*”[tiab] OR “pulmonary adenocarcinoma*”[tiab] OR “non small cell lung carcinoma*”[tiab] OR “large cell lung carcinoma*”[tiab] OR “large cell lung cancer”[tiab] OR “Carcinoma, Non-Small-Cell Lung”[Mesh]

#3 “advanced”[tiab] OR “metastatic” [tiab] OR “Stage IV” [tiab] OR “Stage 4”[tiab]

#4 (#1 AND #2 AND #3)

Restrictions: Clinical Study; Clinical Trial; Clinical Trial, Phase I; Clinical Trial, Phase II; Clinical Trial, Phase III; Clinical Trial, Phase IV; Comparative Study; Controlled Clinical Trial; Evaluation Studies; Multicenter Study; Observational Study; Pragmatic Clinical Trial; Randomized Controlled Trial; 2007 – 2017; Humans

Number of hits: 292

Embase (via embase.com interface) search May 29, 2018

#1 ‘quality of life’/exp OR ‘life quality’:ab,ti OR ‘quality of life’:ab,it

#2 ‘non small cell lung cancer’/exp OR ‘non small cell lung cancer’:ab,ti OR ‘squamous cell carcinoma of the lung*’:ab,ti OR ‘squamous cell lung carcinoma*’:ab,ti OR ‘lung adenocarcinoma*’:ab,ti OR ‘adenocarcinoma of the lung*’:ab,ti OR ‘pulmonary adenocarcinoma*’:ab,ti OR ‘non small cell lung carcinoma*’:ab,ti OR ‘large cell lung carcinoma*’:ab,ti OR ‘large cell lung cancer’:ab,ti

#3 ‘advanced’:ab,ti OR ‘metastatic’:ab,ti OR ‘Stage IV’:ab,ti OR ‘Stage 4’:ab,ti

#4 (#1 AND #2 AND #3)

Restrictions: Human; clinical trial; controlled study; major clinical study; randomized controlled trial; controlled clinical trial; phase 2 clinical trial; questionnaire; phase 3 clinical trial; clinical article; randomized controlled trial (topic); multicenter study; phase 3 clinical trial (topic); phase 2 clinical trial (topic); retrospective study; prospective study; clinical trial (topic); phase 1 clinical trial; 2007-2017; articles

Number of hits: 469

Web of Science (via webofknowledge.com interface) search May 29, 2018

1# ‘quality of life’ OR ‘life quality’

2# ‘non small cell lung cancer’ OR ‘squamous cell carcinoma of the lung*’ OR ‘squamous cell lung carcinoma*’ OR ‘lung adenocarcinoma*’ OR ‘non-small cell lung carcinoma*’ OR ‘adenocarcinoma of the lung*’ OR ‘pulmonary adenocarcinoma*’ OR ‘non small cell lung carcinoma*’ OR ‘large cell lung carcinoma*’ OR ‘large cell lung cancer’

3# ‘advanced’ OR ‘metastatic’ OR ‘Stage IV’ OR ‘Stage 4’

#4 (#1 AND #2 AND #3)

Number of hits: 821
